# Immobilization Techniques for Food-Grade Nuclease P1 and Their Application in Nucleotide Production

**DOI:** 10.3390/foods14040612

**Published:** 2025-02-12

**Authors:** Xiao-Yan Yin, Wei-Zhong He, Yingkun Sheng, Zhong-Hua Yang

**Affiliations:** Xingzhi College, Zhejiang Normal University, Jinhua 321100, China; yinxiaoyan@zjnu.edu.cn (X.-Y.Y.); hewzh@zjnu.cn (W.-Z.H.); yingkunsheng@zjnu.edu.cn (Y.S.)

**Keywords:** nuclease P1, nuclease P1 immobilization, 5′-nucleotide production, enzyme stability, enzyme reusability

## Abstract

Nuclease P1 (NP1) is critical for producing 5′-nucleotides, which are essential flavor enhancers in the food industry. Traditional use of free NP1 is hampered by poor reusability, high costs, and potential residual enzyme protein contamination, compromising product quality. This study introduces a novel immobilization technique using a cross-linking approach with food-grade ion exchange resin AER1 to create NP1@AER1-GA. This method achieves an enzyme activity of 51,015 U/g, with a notable immobilization yield of 67.7%. The immobilized NP1@AER1-GA exhibits significantly enhanced stability and catalytic efficiency while ensuring industrial compatibility and maintaining stringent safety standards. Under optimized conditions, NP1@AER1-GA demonstrates exceptional performance in 5′-nucleotide production, retaining approximately 85% of its initial activity after 10 cycles of reuse. This breakthrough not only boosts the efficiency and sustainability of nucleotide synthesis but also offers a scalable solution for industrial applications, promoting sustainable manufacturing practices within the food industry. By addressing key challenges associated with traditional enzymatic methods, this immobilization technique sets a new benchmark for biocatalyst design in the food processing industry.

## 1. Introduction

Nucleotides are fundamental components of living organisms, serving as the building blocks of nucleic acids and playing pivotal roles in numerous biological processes [1]. Specifically, 5′-nucleotides have garnered significant attention due to their exceptional flavor-enhancing, nutritional, and medicinal properties [2,3,4], finding applications in food additives [5,6], infant milk formula [7], pharmaceuticals [8,9], and animal feeds [10,11].

At present, the production of nucleotides can be achieved through three primary routes: fermentation [12], chemical synthesis [13], and enzymatic hydrolysis of RNA [14]. Among these routes, enzymatic hydrolysis stands out for its efficiency, operational simplicity, high yield, and environmental sustainability [14], making it the preferred route for industrial 5′-nucleotide production.

Enzymatic hydrolysis, particularly when catalyzed by Nuclease P1 (NP1, EC 3.1.30.1), has emerged as a leading technology for producing 5′-nucleotides from RNA [14]. Isolated from *Penicillium citrinum* [15], NP1 is central to this process. With the growing market demand for nucleotides, especially as flavor enhancers, there is an increasing need for NP1. However, challenges related to enzyme stability and recyclability limit its full potential. Immobilizing NP1 on a suitable carrier can enhance its operational stability and reusability, thereby reducing costs and improving productivity and product purity [16,17,18]. Currently, NP1 is predominantly utilized in its free form for batch operations, which requires the addition of fresh enzymes for each reaction batch and prevents the separation of the enzyme post-reaction. This results in high enzyme costs, reduced productivity, and inadequate product purity [19]. This limitation hinders its application in pharmaceuticals and other fine chemicals. Immobilization of NP1 addresses these issues, making it more viable for the food and pharmaceutical sectors [17,20].

The choice of carrier is crucial for effective enzyme immobilization, impacting enzymatic properties, immobilization efficacy, and application suitability. In the food processing industry, selecting a safe and non-toxic carrier material for NP1 immobilization is paramount [5,21]. Food-grade resins offer an ideal solution, combining cost-effectiveness, substantial enzyme loading capacity, robustness, and safety [22]. Given the nature of RNA substrates, minimizing mass transfer resistance within the immobilization matrix is also critical.

This study highlights the advantages of using food-grade resins for NP1 immobilization in 5′-nucleotide production. By developing an efficient covalent immobilization technique, we aim to optimize NP1’s catalytic performance in batch-stirred tank reactors under simulated industrial conditions. This work represents a significant advancement, addressing current limitations while adhering to stringent food safety standards, thus enhancing the industrial application of nucleases for the benefit of both the food and pharmaceutical industries.

## 2. Materials and Methods

### 2.1. Materials

Nuclease P1 (NP1) was produced through fermentation using *Penicillium citrinum* and subjected to preliminary purification via ammonium sulfate precipitation followed by membrane ultrafiltration [17]. The resulting solution, referred to as the NP1 stock solution, exhibited an enzyme activity of 13,253 U/mL. Yeast RNA was sourced from Shanghai Yuanye Biotechnology Co., Ltd. (Shanghai, China). Bovine Serum Albumin (BSA) and glutaraldehyde (GA) were procured from Sigma-Aldrich^®^ (Shanghai, China). Sodium acetate, acetic acid, and other common chemical reagents were acquired from Sinopharm Chemical Reagent Co., Ltd. (Beijing, China). The resins utilized in this study were purchased from three suppliers: Shanghai Anlande Biotechnology Co., Ltd. (Shanghai, China), Tianjin Yunkai Resin Technology Co., Ltd. (Tianjin, China), and Xi’an Lanxiao Technology New Materials Co., Ltd. (Xi’an, China).

### 2.2. Immobilization of NP1 on Resins

To prepare the resins for use, they were first washed according to the manufacturer’s instructions. For activation of the resins, 1.00 g of resin was mixed with 10 mL of a 0.25% glutaraldehyde solution in a 50 mL reaction flask and activated at 25 °C under gentle agitation (60 rpm) for 1.5 h. Following this activation step, the glutaraldehyde-activated resin (Resin-GA) was collected and thoroughly rinsed with deionized water to remove any unbound glutaraldehyde.

For enzyme immobilization, a certain volume of the NP1 stock solution (13,253 U/mL), such as 1 mL, was diluted with acetic acid buffer (10 mM, pH 5.5) to create an 8 mL working solution. Next, 0.2 g of the activated Resin-GA and NP1 working solution were both added into a 50 mL flask. The immobilization reaction proceeded at 25 °C under gentle agitation (60 rpm) for 10 h, allowing NP1 to covalently bind to the resin.

To achieve the optimal immobilization conditions for maximizing immobilized enzyme activity, the enzyme solution volume, the pH of the immobilization system, and the glutaraldehyde activation cross-linking time were further optimized using response surface methodology. The levels of the three variables were as follows: A: Volume of NP1 stock solution 0.75 mL, 1.00 mL, and 1.50 mL; B: pH values of 4.6, 5.0, and 5.4; C: Crosslinking times of 1 h, 1.5 h, and 2 h (for detailed information, see Supplementary Materials, Table S1).

After immobilization, the resulting enzyme–resin complex, designated NP1@Resin-GA, was washed with acetic acid buffer (10 mM, pH 5.5) at 4 °C to remove unbound enzymes and impurities. Surface moisture on the NP1@Resin-GA was gently blotted using filter paper, and the sample was stored at 4 °C for future use.

### 2.3. Enzymatic Properties of NP1@Resin-GA

To assess the performance of NP1 immobilized on resin, a comprehensive assessment of its enzymatic properties was conducted. This included examining the optimal reaction temperature and pH for NP1@Resin-GA, as well as evaluating their thermal stability, pH stability, and storage stability. Kinetic parameters were also determined using the Michaelis–Menten equation to estimate enzyme efficiency and substrate affinity [17,23]. The study systematically compared the characteristics of free NP1 with those of the immobilized enzyme, NP1@Resin-GA, to highlight any improvements in operational conditions and stability. This approach provides a thorough understanding of how immobilization affects enzyme performance, offering insights into the potential advantages for industrial applications.

### 2.4. Application of NP1@AER1-GA in 5′-Nucleotides Production

The catalytic efficiency of NP1@AER1-GA in producing 5′-nucleotides through RNA hydrolysis was evaluated using established methods [17,19,24]. To comprehensively assess the catalytic performance of NP1@AER1-GA, its ability to hydrolyze RNA was investigated across a wide range of RNA concentrations. Specifically, RNA concentrations were increased from 3% to 10% (equivalent to 30 to 100 g/L) at 65 °C and pH 5.5 to evaluate how substrate concentration affects the enzyme’s activity. This approach allowed us to determine the optimal RNA concentration for maximal catalytic efficiency and to understand the enzyme’s performance under different substrate levels. By systematically varying the RNA concentration, this study provides detailed insights into the catalytic capabilities of NP1@AER1-GA, highlighting its potential for efficient nucleotide production in industrial applications.

To evaluate the reusability and repeated-use performance of NP1@AER1-GA, repeated batch operations were conducted in a batch-stirred tank reactor (BSTR). For each cycle, 0.5 g of NP1@AER1-GA with an enzyme activity of 50,901 U/g was added to the BSTR, along with 1000 mL of a 60 g/L RNA solution at pH 5.5. The hydrolysis reaction proceeded for 4 h at 65 °C under continuous stirring at 100 rpm. Upon completion of each batch, the reaction mixture was filtered. The recovered NP1@AER1-GA was thoroughly rinsed with deionized water, and surface moisture was gently removed using filter paper before reuse in subsequent cycles. The filtrate from each batch was collected, and the residual RNA concentration was quantified by spectrophotometry. This protocol allowed for a detailed assessment of the enzyme’s performance over multiple cycles, providing insights into its operational stability and efficiency in nucleotide production.

### 2.5. Analysis Method

#### 2.5.1. Determination of Protein-Loading Capacity of Resins

Measure out 0.1 g of GA-activated resins and transfer each portion into separate 10 mL capped vials. Introduce 5 mL of a BSA solution, with a concentration of 100 μg/mL, to each vial, seal them tightly, and incubate at a temperature of 25 °C while shaking at 80 rpm for a duration of 2 h. Upon completion of the incubation period, gently pour off the liquid above the settled resin and rinse the resin multiple times. Gather the rinse liquids and amalgamate them with the original supernatant. Assess the BSA content in this combined liquid utilizing the Coomassie Brilliant Blue technique [25]. Subsequently, compute the ratio of BSA loaded onto the resin based on the determined concentration using the formula provided as equation (Equation (1)):

(1) $$\mathrm{Protein}\;\mathrm{Adsorption}\;\mathrm{Capacity}=\left(1-\frac{\mathrm{BSA}\;\mathrm{content}\;\mathrm{in}\;\mathrm{the}\;\mathrm{solution}\;\mathrm{after}\;\mathrm{loading}}{\mathrm{BSA}\;\mathrm{content}\;\mathrm{in}\;\mathrm{the}\;\mathrm{solution}\;\mathrm{before}\;\mathrm{loading}}\right)\times 100\%$$

#### 2.5.2. Assay of Nuclease P1 Activity

The NP1 activity was evaluated by its proficiency in catalyzing RNA’s breakdown into 5′-nucleotides, substances that exhibit a distinctive absorbance peak at 260 nm following the precipitation of RNA. An enzyme unit is defined as the amount of enzyme causing an increase in optical density of 1.0 at 260 nm each minute under optimal conditions for catalysis [17,26,27].

To assess the activity of free NP1 and immobilized NP1@AER1-GA, the following procedure was applied: A defined volume of a 3% RNA solution (0.95 mL for evaluating free NP1 or 3 mL for NP1@AER1-GA) was warmed in a temperature-controlled water bath at 80 °C for 5 min. Following this, 0.05 mL of the free NP1 (or 0.01 g of NP1@AER1-GA) was introduced into the RNA solution, and the resulting mixture was incubated at 80 °C for an additional five-minute period. Subsequently, 1 mL of nucleic acid precipitating agent—a solution composed of 0.25% ammonium molybdate and 2.5% perchloric acid—was added into the reaction mixture, which was then thoroughly mixed using a vortex. This mixture was promptly moved to an ice bath and held there for 10 min. It was then subjected to centrifugation, after which the supernatant was diluted to a predetermined factor, and its absorbance was measured at 260 nm.

The enzymatic activities for both the free NP1 and the NP1@AER1-GA were, respectively, computed using equations (Equations (2) and (3)). The immobilization efficiency of NP1 activity, denoted as Yield, was determined using equation (Equation (4)).

For the enzyme activity of free NP1, denoted as EA (U/mL), the following equation is used:

(2) $$\mathrm{EA}=\frac{\left(A-A_{0}\right)\times f\times 2\times n}{0.05\times 5}$$

The enzyme activity of NP1@AER1-GA, represented as EA_imm_ (U/g), is calculated by:

(3) $${\mathrm{EA}}_{\mathrm{imm}}=\frac{\left(A-A_{0}\right)\times 6\times n}{0.01\times 5}$$

The efficiency of immobilization for NP1@AER1-GA, expressed as a percentage yield, is given by:

(4) $$\mathrm{Yield}\;\mathrm{of}\;\mathrm{immobilized}\;\mathrm{NP}1=\frac{{\mathrm{EA}}_{\mathrm{imm}}}{{\mathrm{EA}}_{0}-{\mathrm{EA}}_{1}}\times 100$$

where A: the absorbance from the experimental samples; A_0_: the absorbance of the control without enzyme; f: the dilution factor applied to the enzyme solution; n: the dilution ratio of the centrifuged supernatant from the reaction mix; EA_imm_: the activity of NP1@AER1-GA, U/g; EA_0_: the total activity of free NP1 before immobilization, U; and EA_1_: the residual activity of free NP1 post-immobilization, U.

#### 2.5.3. RNA Quantitative Analysis

RNA quantification was performed via spectrophotometric analysis [17]. The molar absorptivity ε, indicating the alteration in absorbance due to the depletion of 1 g/L RNA within the reaction environment, was computed using Equation (5). Experimental findings alongside the regression line are detailed in the Supplementary Materials (Figure S1). It was established that the adjusted molar absorptivity is ε = 19.63. Leveraging this figure, one can ascertain the quantity of RNA processed at any point during the NP1-mediated reaction [28].

Molar absorptivity equation

(5) ε=A/ω

Note: A denotes the absorbance from the enzymatic reaction system; C signifies the concentration of RNA consumed within this system.

The reaction velocities of NP1@AER1-GA and free NP1 across different RNA concentrations were modeled using the molar absorptivity. Ultimately, the kinetic parameters for the Michaelis–Menten equation (Equation (6)) pertaining to NP1@AER1-GA and free NP1 were regressed through the Levenberg–Marquardt algorithm [29].

(6) $$v={\frac{K_{m}\cdot \left[S\right]}{K_{m}+\left[S\right]}}_{\max}$$

#### 2.5.4. Statistical Analysis

All experiments were conducted in three replicates. The data reflect the average of these replicate values. The associated standard deviation was computed. Statistical assessments were performed using Excel (2021) and OriginPro (2021 9.8.0.200).

## 3. Results and Discussion

### 3.1. Immobilization of NP1@Resin-GA

#### 3.1.1. Resin Selection

Choosing an appropriate resin is critical for the successful immobilization of NP1, especially when intended for food processing applications. This study evaluated various food-grade resins, including epoxy resins, ion-exchange resins, and macroporous adsorption resins, to identify the most suitable carrier for NP1 immobilization. Table 1 summarizes the types of resins, their functional groups, protein adsorption capacities, and NP1 immobilization efficiencies. While the adsorption capacity for the model protein BSA was similar across different resins, significant variations were observed in NP1 immobilization efficiency. Enzyme activity after immobilization on certain resins (ER, MR1, MR2, CR1, CR2, CR3, CGR) was notably low, likely due to interactions between the resin’s functional groups—such as carboxyl, cyano, and ester groups—and NP1’s active site. In contrast, resins AR2, EAR, AER1, and AER2 demonstrated superior immobilization efficiency, with AER1 exhibiting the highest enzyme activity post-immobilization. The best performance of AER1 can be attributed to its functional groups, porosity, and internal pore size, which facilitate optimal interaction between the resin and NP1. These factors also enhance substrate transport performance, making AER1 particularly suitable for binding NP1 while ensuring efficient RNA hydrolysis [30,31]. Consequently, AER1 was selected as the immobilization carrier, achieving an immobilized enzyme activity of 35,132 U/g, surpassing previously reported results [31].

#### 3.1.2. Optimization of NP1 Immobilization @ Resin-GA

**Figure 1:**
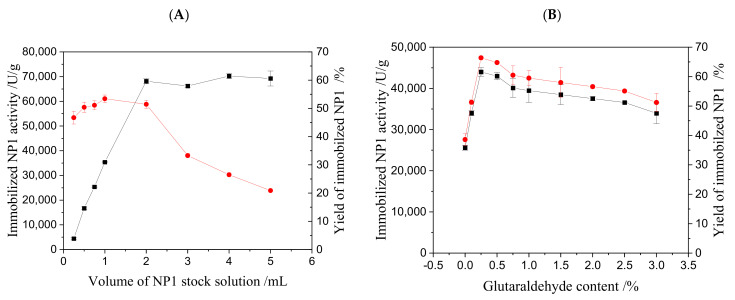

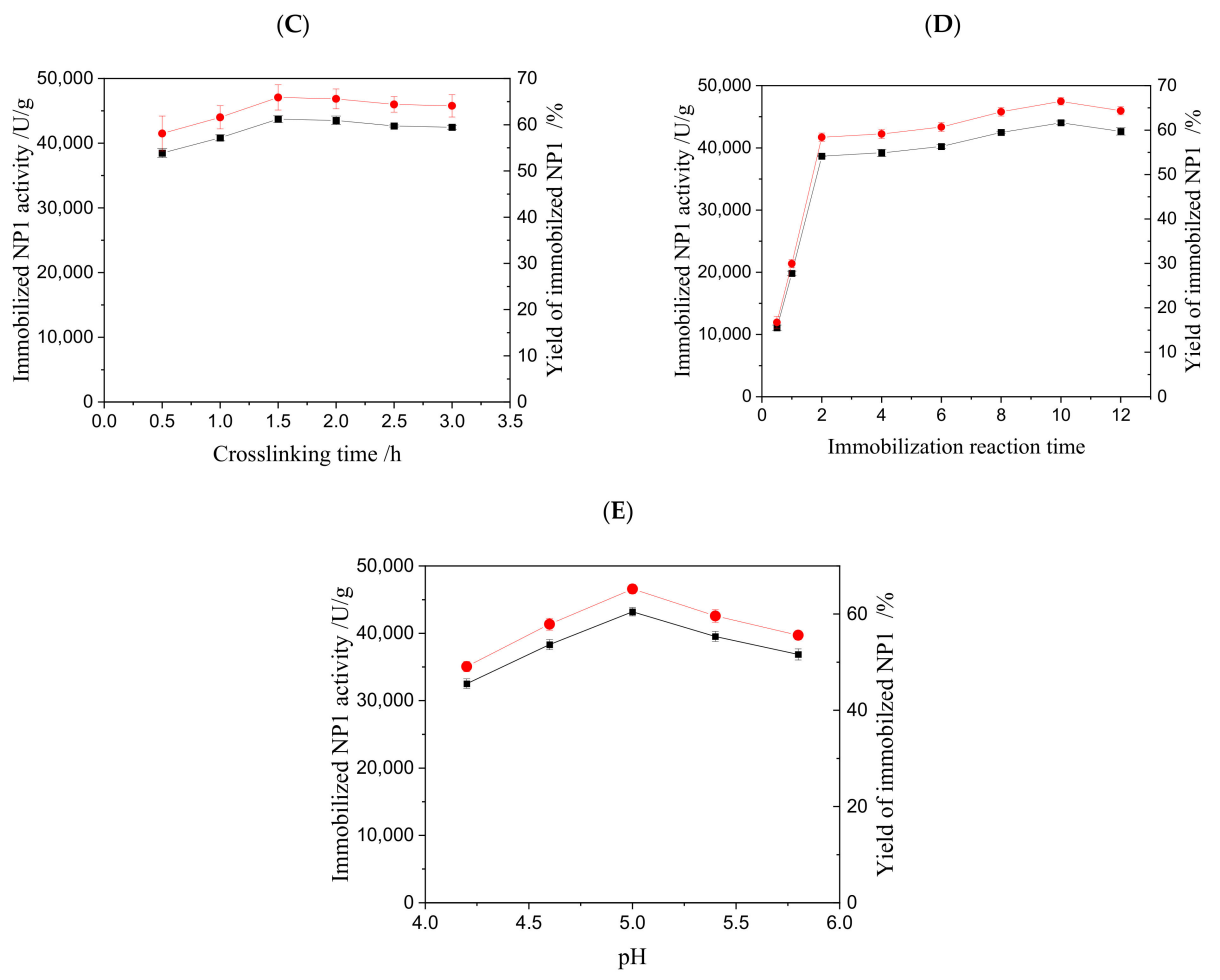
Influence of immobilization conditions on the efficiency of NP1 immobilization @AER1-GA resin. (**A**) Enzyme amount; (**B**) glutaraldehyde content; (**C**) glutaraldehyde crosslink time; (**D**) immobilization reaction time; (**E**) pH. Condition: Activation of the resins, 1.00 g of resin, 10 mL of 0.25% glutaraldehyde, 1.5 h. Enzyme immobilization: 1 mL of NP1 stock solution, 0.2 g of the activated Resin-GA, pH 5.5 for 10 h. ■ Activity; ● Yield.

To achieve optimal immobilization conditions, the effects of various factors on immobilization efficiency were investigated, focusing on immobilized NP1 activity and yield. Key parameters included the amount of NP1 stock solution, concentration of crosslinking agent glutaraldehyde (GA), activation time of GA to AER1, immobilization reaction time, and pH of the immobilization system. The results are presented in Figure 1.

The effect of enzyme quantity on the NP1 immobilization is presented in Figure 1A. Increasing the amount of NP1 stock solution initially enhances immobilized enzyme activity until it reaches a peak at approximately 1 mL (1656.6 U/mL of NP1 in immobilization solution). Beyond this point, activity decreases, likely due to the carrier reaching its maximum enzyme-carrying capacity. This trend is similar to Shi’s report in their immobilization NP1 on paper cellulose [20]. Figure 1B shows the effect of GA concentration on the NP1 immobilization efficiency. A GA concentration of 0.25% yielded the best immobilization efficiency, which is similar to Shi’s result [32]. Higher concentrations reduced efficiency, possibly due to excessive aldehyde groups reacting with enzyme amino acid side chains or active sites, leading to decreased enzyme activity. The effect of the activation time of GA on the NP1 immobilization is presented in Figure 1C. An activation time of 1.5 h resulted in optimal immobilization efficiency. Longer activation times may over-activate resin functional groups, hindering subsequent reactions with the enzyme. The effect of immobilization reaction time on the NP1 immobilization is presented in Figure 1D. After 2 h, no significant changes in immobilized enzyme activity were observed, indicating that this duration is sufficient for optimal immobilization. Figure 1E shows the effect of pH on the NP1 immobilization. The highest immobilized enzyme activity and yield were achieved at pH 5.0, reflecting the optimal reaction environment for enzyme–crosslinking agent interactions [20].

**Figure 2:**
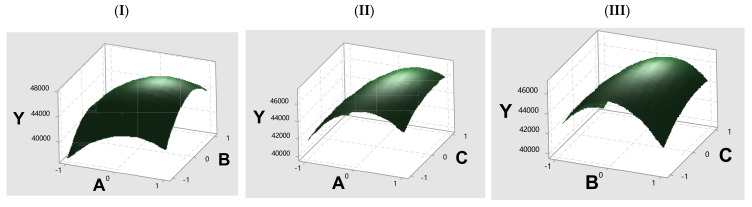
Plot the response surface of immobilized enzyme activity vs. various factors. (**I**) Immobilized enzyme activity vs. enzyme solution volume and pH; (**II**) immobilized enzyme activity vs. enzyme solution volume and Crosslinking time; (**III**) immobilized enzyme activity vs. pH and Crosslinking time. Axis labels: Y: Immobilized enzyme activity; A: Volume NP1 stock solution 0.75 mL, 1.00 mL, and 1.50 mL; B: pH 4.6, 5.0 and 5.4; C: Crosslinking time 1 h, 1.5 h, and 2 h.

Based on these findings, three main factors influencing immobilization efficiency were identified: enzyme quantity, immobilization reaction pH, and GA activation time. Response surface methodology (RSM) using Minitab (17.1.0) was employed to further optimize these conditions. The Box–Behnken experimental design and analysis are detailed in Supplementary Materials (Tables S1–S3), with response surface plots shown in Figure 2. The prediction conditions were 1.14 mL of NP1 stock solution, pH 5.05, and 1.56 h activation time, reaching an immobilized activity of 50,901 U/g with a 67.6% immobilization yield. Based on this prediction, optimized conditions included 1.14 mL of NP1 stock solution (corresponding to a working concentration of 1889 U/mL), pH 5.1, and a crosslinking time of 1.6 h was applied to immobilize NP1@AER1-GA. Under these conditions, NP1@AER1-GA achieved an enzyme activity of 51,015 U/g with a 67.7% immobilization yield, closely matching the predicted value. This validation confirms the effectiveness of the optimization model and the reasonableness of the chosen immobilization conditions.

### 3.2. Enzymatic Properties of NP1@AER1-GA

**Figure 3:**
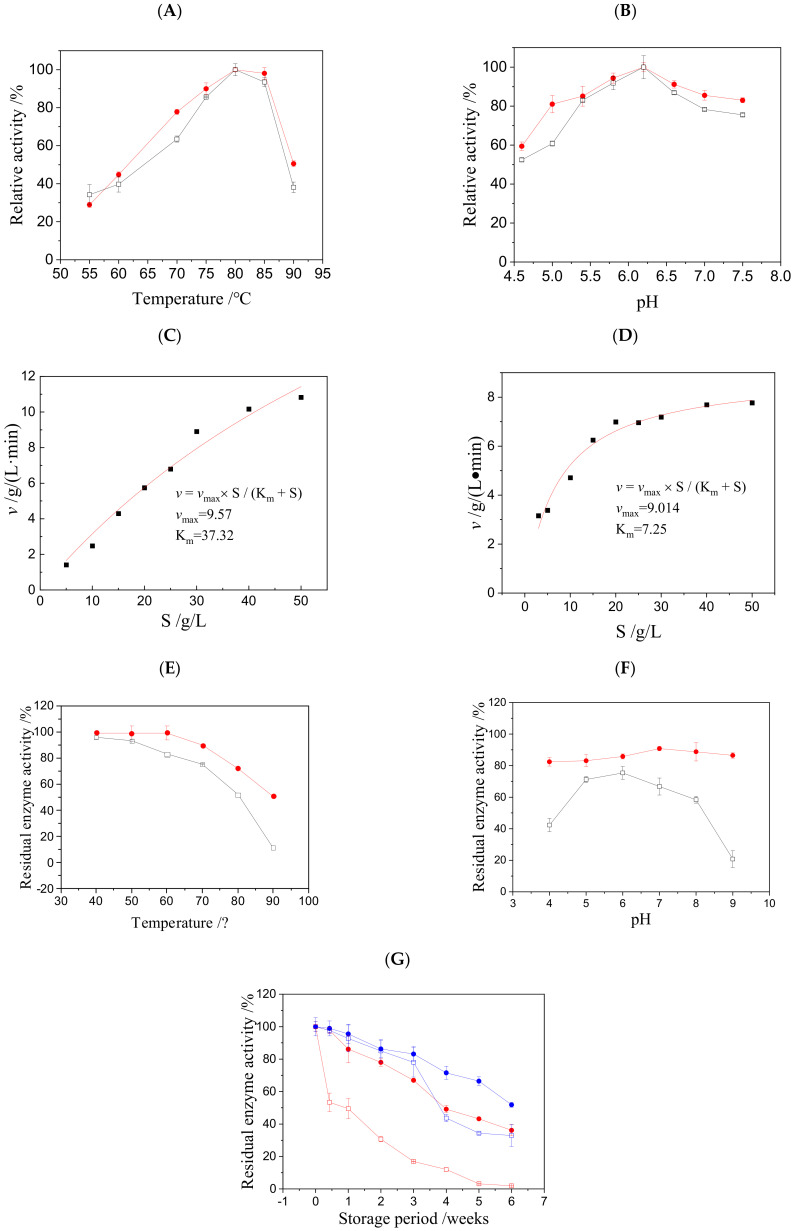
Enzymatic properties of NP1@AER1-GA and free NP1. (**A**) Effect of temperatures on enzyme activity; (**B**) effect of pH on enzyme activity properties; (**C**) kinetic parameters fitting of NP1@AER1-GA; (**D**) kinetic parameters fitting of free NP1; (**E**) thermal stability; (**F**) pH stability; (**G**) storage stability. ●: NP1@AER1-GA, □: free NP1 (for (**A**,**B**,**E**,**F**)); ■: experimental data, ―: fitted curve (for (**C**,**D**)); ●: NP1@AER1-GA at 4 °C, ●: NP1@AER1-GA at room temperature, □: free NP1 at 4 °C, □: free NP1 at room temperature (for (**G**)).

The enzymatic properties of NP1@AER1-GA were rigorously analyzed, focusing on optimal temperature and pH, kinetic parameters, and stability. For comparison, the corresponding traits of free NP1 were also examined. All results are illustrated in Figure 3.

#### 3.2.1. Optimal Reaction Temperature and pH

The results, presented in Figure 3A,B, indicate that the enzymatic behavior of NP1@AER1-GA closely mirrors that of free NP1. This suggests that immobilization onto AER1-GA does not significantly alter the intrinsic enzymatic characteristics of NP1, aligning with previous studies on NP1 immobilization using various carriers [17,20]. However, a notable distinction is that NP1@AER1-GA exhibits an extended range of suitable temperatures and pH values compared to free NP1. This broader operational range implies enhanced practical efficiency for NP1@AER1-GA in manufacturing processes.

#### 3.2.2. Kinetic Analysis

To evaluate the kinetic parameters, the Michaelis–Menten equation was applied to model the reaction kinetics for both NP1@AER1-GA and free NP1. The kinetic parameters were determined based on the initial velocity of reactions at different RNA concentrations. The results are presented in Figure 3C,D. NP1@AER1-GA has a K_m_ value of 37.32 g/L, while free NP1 has a lower K_m_ of 7.25 g/L. The *v*_max_ for NP1@AER1-GA is 9.57 g/(L·min), slightly higher than the 9.01 g/(L·min) observed for free NP1. The increased K_m_ value for NP1@AER1-GA can be attributed to two factors: changes in the enzyme’s molecular structure during immobilization, and increased substrate transfer resistance, which reduces the distribution factor between the matrix and liquid phase [17,33]. Comparatively, NP1 immobilized on other carriers such as nanoparticles (K_m_ = 8.06 mg/mL), cellulose (K_m_ = 9.14 mg/mL), DEAE cellulose (K_m_ = 27.21 mg/mL), paper-based cellulose (K_m_ = 5.72 mg/mL, *v*_max_ = 0.16 mg/(mL·min)), and macroporous absorbent resins (K_m_ = 18.125 mg/mL, *v*_max_ = 443.95 U/(mL·min)) shows varying levels of catalytic efficacy [20,26,32,34]. Despite these differences, NP1@AER1-GA demonstrates superior catalytic efficiency.

#### 3.2.3. Stability Analysis

Significantly, NP1@AER1-GA exhibits enhanced stability compared to free NP1 in terms of thermal, pH, and storage stability. As presented in Figure 3E, NP1@AER1-GA shows markedly improved thermal stability relative to free NP1, which was also found by Li [34]. As shown in Figure 3F, NP1@AER1-GA maintains activity across a broader pH spectrum due to the presence of abundant -CH₂N⁺(CH₃)₃ clusters on the AER1-GA surface, which provide stabilizing effects [26]. Regarding storage stability (results presented in Figure 3G), while free NP1 retains satisfactory stability at 4 °C, NP1@AER1-GA achieves notably superior long-term storage. After 6 weeks at 4 °C, NP1@AER1-GA retained over 60% of its initial activity, demonstrating a significant enhancement in storage robustness post-immobilization. It is comparable with our previous results of NP1@CSMs-GA, which could retain more than 65% initial activity after 49 d of storage [17].

In summary, NP1@AER1-GA not only preserves the inherent enzymatic properties of NP1 but also extends its operational range and enhances stability, making it a promising candidate for industrial applications.

### 3.3. Production of 5′-Nucleotides with NP1@AER1-GA

The production of 5′-nucleotides through RNA hydrolysis catalyzed by free NP1 in batch-stirred tank reactors (BSTRs) is a common practice in the food industry [19]. However, this process requires fresh NP1 for each batch, leading to significant enzyme consumption and increased production costs. Moreover, residual deactivated NP1 left in the reaction mixture can compromise the purity and quality of the final product. Immobilized NP1 offers a compelling solution by enabling easy removal from the reaction mixture and facilitating multiple-use cycles, thereby enhancing both efficiency and cost-effectiveness [17]. To evaluate the production performance of NP1@AER1-GA, we investigated its ability to hydrolyze RNA at various RNA concentrations and assessed its reusability in a BSTR.

#### 3.3.1. NP1@AER1-GA Catalysis RNA Hydrolysis Performance

**Figure 4:**
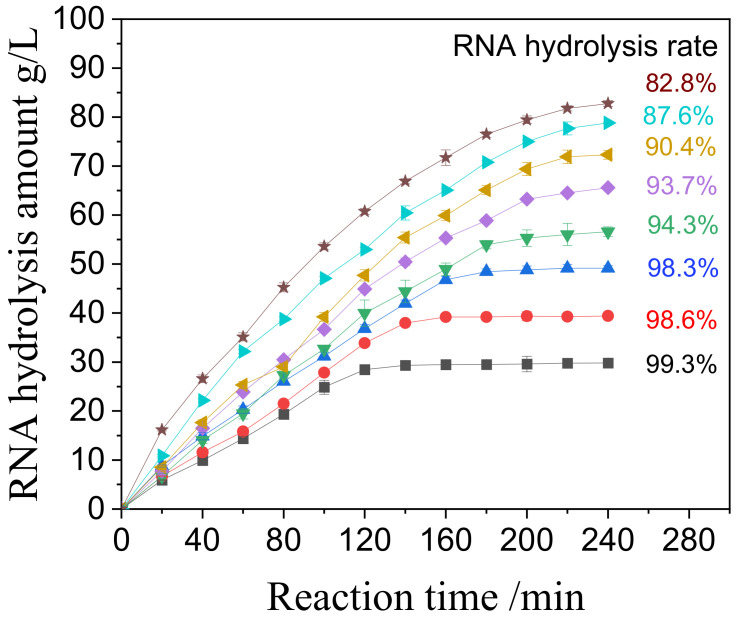
Time course curves of hydrolysis RNA catalyzed by NP1@AER1-GA at various concentrations of RNA (from 3% to 10%, i.e., 30 g/L to 100 g/L) at 65 °C and pH 5.5. ■: 3%, ●: 4%, ▲: 5%, ▼: 6%, ◆: 7%, ◄: 8%, ►: 9%, ★: 10%.

RNA concentrations ranging from 3% to 10% (equivalent to 30 to 100 g/L) were tested to determine the optimal substrate concentration for practical application. The time course of RNA hydrolysis catalyzed by NP1@AER1-GA is illustrated in Figure 4.

The results show that NP1@AER1-GA exhibits robust catalytic activity across all tested concentrations. It is comparable to our previous work on NP1@CSMs-GA, where the catalysis activity could keep more than 80% with 3% to 9% RNA [17]. At 5% RNA concentration, complete hydrolysis was achieved within 160 min with a yield exceeding 98%. At 8% RNA concentration, equilibrium was reached within 220 min, achieving a yield of over 90%. Even at the highest concentration of 10%, hydrolysis was nearly complete within 240 min, with a yield of 82%. Considering both reaction time and hydrolysis yield, an 8% RNA concentration appears optimal for industrial applications, balancing efficiency and throughput.

#### 3.3.2. NP1@AER1-GA Reusability

**Figure 5:**
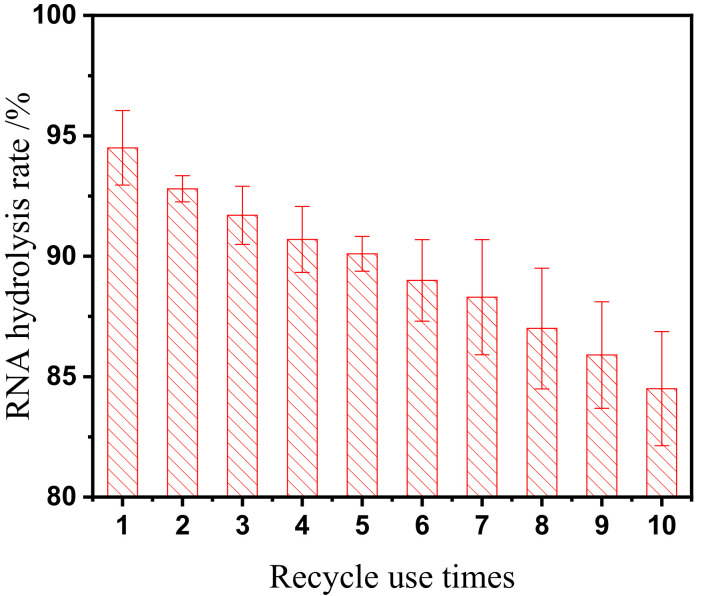
Recycle use times impact on NP1@AER1-GA-GA-catalyzed RNA hydrolysis efficiency at 65 °C and pH 5.5 with 60 g/L RNA.

Figure 5 presents the results of repeated use of NP1@AER1-GA in a BSTR. Despite a gradual decline in efficiency with increasing reuse cycles, NP1@AER1-GA retained 84.5% of its initial activity after 10 cycles. This reduction in efficiency may be attributed to the elevated reaction temperature (65 °C) and minor losses during handling and cleaning between cycles. In contrast, free nuclease P1 cannot be reused, limiting it to single-use applications.

Given that NP1@AER1-GA retains approximately 85% of its initial activity after 10 cycles, it demonstrates stable operational characteristics and reusable performance. Considering the enzyme activity yield of 67.7% achieved through immobilization, the effective utilization value of nuclease P1 increases by 606% after just 10 cycles, highlighting the significant economic benefits of using immobilized enzymes. The reusability of this immobilization technology is comparable to the results of NP1@CSMs-GA [17].

NP1@AER1-GA not only preserves the inherent enzymatic properties of NP1 but also extends its operational range and enhances stability, making it a promising candidate for industrial nucleotide production. The combination of high catalytic efficiency and reusability significantly reduces production costs and improves product purity, underscoring the advantages of immobilized enzymes in biotechnological processes.

## 4. Conclusions

This study advanced the immobilization of NP1 for 5′-nucleotide production, utilizing a cross-linking approach with food-grade AER1 resin. The resulting NP1@AER1-GA exhibited superior operational stability and catalytic efficiency, achieving an enzyme activity of 51,015 U/g with an immobilization yield of 67.7%. The broader operational range in terms of temperature and pH also signifies practical advantages for manufacturing processes. However, the immobilization process led to an increased K_m_ value, indicating higher substrate transfer resistance, which could be attributed to changes in enzyme shape or matrix diffusion limitations. Compared to free NP1, NP1@AER1-GA demonstrated enhanced thermal, pH, and storage stability, exhibiting superior catalytic performance. NP1@AER1-GA has excellent reusability performance, maintaining approximately 85% of its initial activity after 10 recycling cycles. Unlike the single-use limitation of free enzymes, this reusability drastically reduces costs and improves product purity.

One limitation of this study is that it further enhances the transfer efficiency of substrate RNA in immobilized NP1, which is a key factor affecting the catalytic rate. However, despite this limitation, the findings offer valuable insights into the potential applications of NP1 immobilization under controlled conditions. The study focused on batch-stirred tank reactors; continuous flow systems may offer further productivity gains and cost reductions.

To mitigate these challenges, future work should explore techniques that reduce substrate transfer resistance. Expanding the application to continuous flow reactors may provide insights into more efficient processing. Additionally, exploring new hypotheses about how different carrier properties affect enzyme function could lead to innovations in biocatalysis technology, potentially broadening industrial applications and contributing to sustainable manufacturing practices.

## Figures and Tables

**Table 1:** Immobilization effect of NP1 by various resins.

Resin Type *	Functional Group	Protein Adsorption Capacity/µg/g_resin_	Enzyme Activity Adsorption Capacity/U/g_resin_	Immobilized Enzyme Activity/U/g
ER	-CH(O)CH_2_	9742.1	22,700.0	434.7
AR1	-NH_2_	10,337.9	50,066.6	542.3
AR2	-NH_2_	9589.8	152,990.0	27,916.2
EAR	-CH(NH_2_)CH(O)CH_2_	9628.9	133,405.0	32,026.6
AER1	-CH_2_N^+^(CH_3_)_3_Cl^−^	9686.4	155,008.3	35,131.7
AER2	-CH_2_N^+^(CH_3_)_3_Cl^−^	9520.9	119,243.3	28,713.9
MPAR1	-C18	10,407.6	89,035.0	124.1
MPAR2	-COOCH_3_	10,465.5	40,400.0	126.7
CR1	-N(CH_2_COONa)_2_	10,285.2	107,416.6	425.5
CR2	-COONa	12,310.2	41,983.3	367.8
CR3	-CH(CH_2_COONa)_2_	11,776.3	46,400.0	918.9
CGR	-CH(CN)_2_	8750.8	63,080.0	1496.9

* Note: ER is an epoxy resin, AR1 and AR2 are amino resins, EAR is a resin with both amino and epoxy groups, AER1 and AER2 are anion exchange resins, MPAR1 and MPAR2 are macroporous adsorption resins, CR1, CR2, and CR3 are cation exchange resins, and CGR is a resin with a cyanide group.

## Data Availability

The original contributions presented in this study are included in the article/Supplementary Material. Further inquiries can be directed to the corresponding author.

## Supplementary Materials

The following supporting information can be downloaded at: https://www.mdpi.com/article/10.3390/foods14040612/s1, Figure S1: The relationships between absorbance at 260 nm and complete hydrolysis of RNA with different concentrations; Table S1: Factors-level table of Box–Behnken design for response surface optimization of NP1; Table S2: Box–Behnken Design experiment results; Table S3: Analysis of variance for response surface regression.
