# Supplementary material for: Immobilization Techniques for Food-Grade Nuclease P1 and Their Application in Nucleotide Production
Source: Foods. 2025 Feb 12;14(4):612. doi: 10.3390/foods14040612 (PMC11854549; doi:10.3390/foods14040612)

Supplementary Materials

Immobilization Techniques for Food-Grade Nuclease P1 and Their Application in Nucleotide Production

Xiao-Yan Yin, Yingkun Sheng, Zhong-Hua Yang*

Xingzhi College, Zhejiang Normal University, Jinhua 321100, China

*Corresponding author

Zhong-Hua Yang

E-mail: yangzh@zjnu.edu.cn

ORCID: 0000-0001-8341-4775

Table S1 Factors-level table of Box-Behnken design for response surface optimization of NP1 immobilization conditions

Factor	Level		
	-1	0	1
A: Volume NP1 stock solution /mL	0.75	1.00	1.50
B: pH	4.6	5.0	5.4
C: Crosslinking time /h	1.00	1.50	2.00

Table S2 Box-Behnken Design experiment results

Run Order	A*	B	C	Y
1	1	0	1	45756.9
2	0	-1	1	40296.3
3	0	0	0	51826.0
4	0	1	-1	41676.2
5	0	0	0	51826.0
6	-1	0	-1	42867.9
7	0	-1	-1	41729.7
8	1	1	0	44444.4
9	0	0	0	47826.0
10	1	-1	0	41800.0
11	-1	0	1	40142.8
12	1	0	-1	44222.2
13	-1	1	0	37866.7
14	0	1	1	45969.3
15	-1	-1	0	36528.5

*note: A: Enzyme solution volume /mL; B: pH; C: Crosslinking time /h; Y: Immobilized NP1 activity /U/g.

Table S3 Analysis of variance for response surface regression

Source	DF	Adj SS	Adj MS	F-Value	P-Value
Model	9	265207446	29467494	9.19	0.012
Linear	3	56136120	18712040	5.84	0.043
A	1	44262759	44262759	13.81	0.014
B	1	11525041	11525041	3.59	0.116
C	1	348320	348320	0.11	0.755
Square	3	195910112	65303371	20.37	0.003
A*A	1	83363679	83363679	26.00	0.004
B*B	1	115013420	115013420	35.87	0.002
C*C	1	22959304	22959304	7.16	0.044
2-Way Interaction	3	13161214	4387071	1.37	0.353
A*B	1	426540	426540	0.13	0.730
A*C	1	4536474	4536474	1.42	0.288
B*C	1	8198201	8198201	2.56	0.171
Error	5	16029787	3205957		
Lack-of-Fit	3	5363120	1787707	0.34	0.806
Pure Error	2	10666667	5333333		
Total	14	281237232			

Model Summary

S	R-sq	R-sq(adj)	R-sq(pred)
1790.52	94.30%	84.04%	60.95%

Regression Equation in Uncoded Units

Y = 50493 + 2352 A + 1200 B + 209 C - 4752 A*A - 5581 B*B - 2494 C*C + 327 A*B + 1065 A*C + 1432 B*C

Response Optimization: Y

Variable	Setting
A	0.272727
B	0.131313
C	0.131313

Response	Fit	SE Fit	95% CI	95% PI
Y	51901	1002	(48325, 53477)	(45627, 56175)

Figure S1. The relationships between absorbance at 260 nm and complete hydrolysis of RNA with different concentrations

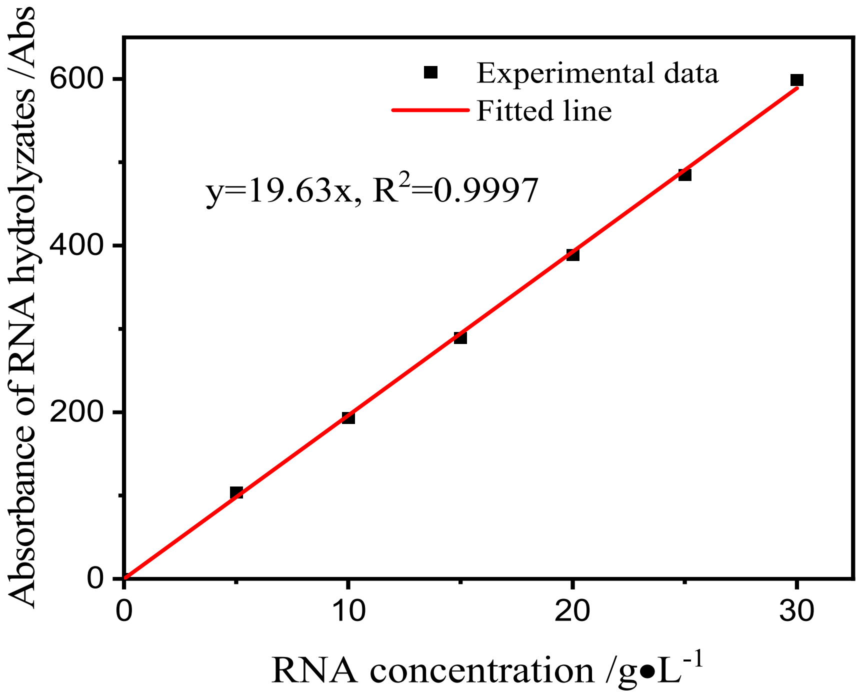

Supplement: Supplementary file 1 [file foods-14-00612-s001.zip › foods-3451437-supplementary.pdf]
